# Having Plans for the Future in Very Old People

**DOI:** 10.1177/00914150241231189

**Published:** 2024-02-11

**Authors:** Martin Bergfrid, Yngve Gustafson, Håkan Littbrand, Birgitta Olofsson, Bodil Weidung

**Affiliations:** 1 Department of Community Medicine and Rehabilitation, Geriatric Medicine, 8075 Umeå University, Umeå, Sweden; 2 Department of Nursing, 8075 Umeå University, Umeå, Sweden; 3 Department of Surgical and Perioperative Science, Orthopaedics, 8075 Umeå University, Umeå, Sweden; 4 Department of Public Health and Caring Sciences, Geriatric Medicine, Uppsala University, Uppsala, Sweden

**Keywords:** aged 80 and over, future perception, gerontology, optimism, plans for the future, survival

## Abstract

This study aimed to investigate the prevalence of having plans for the future among very old people and the factors associated with having such plans. A longitudinal population-based study with home visits for 85-, 90-, and ≥95-year-old participants in Sweden and Finland was used. Multivariate logistic regression and Cox proportional-hazards regression models with a maximum 5-year follow-up period were used. The prevalence of having plans for the future was 18.6% (174/936). More men than women and more people living in Sweden than in Finland had plans for the future. In multivariate models, having plans for the future was associated with speaking Swedish, being dentate, and living in the community in the total sample; speaking Swedish and being dentate among women; and speaking Swedish, having a lower Geriatric Depression Scale score, and urban residence among men. Having plans for the future was associated univariately, but not multivariately, with increased survival.

Life expectancy is increasing dramatically, and more people are entering very old age (≥80 years) (United Nations, Department of Economic and Social Affairs, Population Division, 2015). Despite this development, little is known about very old peoples’ attitudes toward the future. Older people may contemplate and relate to their futures in a different manner than do young and middle-aged people, due to their shorter remaining life expectancies. Growing old is also generally associated with physical, social, and psychological losses, which may influence well-being and attitudes toward the future. Having plans for the future is an expression of a positive future perception and may be an indicator of positive outcomes, including well-being and survival (Hoppmann et al., 2017; Kotter-Grühn & Smith, 2011; Pitkälä et al., 2004). In contrast, the presence of negative thoughts about the future may be associated with lower life satisfaction in older people (Corlett & MacLeod, 2021).

Several studies have shown that the proportion of people who have plans for the future decreases with age (Eloranta et al., 2012; Fagerström, 2010; Kotter-Grühn & Smith, 2011; Pitkälä et al., 2004). However, those studies included few participants aged ≥85 years, and whether this trend persists among very old people remains uncertain. In addition, the prevalence of having plans for the future among very old people remains largely unknown. Factors influencing the presence of plans for the future also have not been investigated extensively in this age group. Some researchers have reported that more men than women have plans for the future (Fagerström, 2010; Kotter-Grühn & Smith, 2011; Tilvis et al., 2012), whereas another group found no gender difference after adjusting for age and living arrangements (Eloranta et al., 2012). To our knowledge, factors associated with having plans for the future have not been examined extensively among women and men separately. Furthermore, differences related to cultural and linguistic factors may exist; among 65- and 75-year-old people, fewer Swedish-speaking Finns than Finnish-speaking Finns and Swedish people had plans for the future (Fagerström, 2010), but whether such an association is present among very old people remains unknown.

Several studies have shown that a positive perception of the future is associated with longevity in younger and middle-aged people (Brummett et al., 2006; Giltay et al., 2004; Maruta et al., 2000; Tindle et al., 2009). The association between having plans for the future and survival in older people was investigated using the Life Orientation Scale (LOS, which measures positive life orientation and optimism) in one study; the researchers found that this association was significant in analyses adjusted for gender, age, and the remaining LOS components (Tilvis et al., 2012). Further research is needed to determine whether having plans for the future is associated with increased survival in very old people, with adjustment for potential confounders, such as medical and social conditions.

## Aims

The aims of this study were to examine the extent to which very old people have plans for the future and to identify factors associated with having such plans. Differences related to gender, country of residence, and language, and the association of having plans for the future with survival, were investigated in the representative study sample of very old people.

## Methods

### Study Design

The data used in this study were collected as part of the Umeå 85+/Gerontological Regional Database (GERDA) study, a population-based, cross-sectional, longitudinal cohort study for which inclusion criteria were based on age and place of residence. Participant recruitment was initiated in 2005, 2007, 2010, and 2012. In addition, those who had participated in 2005 and 2007 were asked to participate again in 2010 and 2012, respectively. Population registers were obtained from the National Tax Board of Sweden and the Finnish Population Register Center. Using a randomized staring point, every other 85-year-old, all 90-year-old, and all ≥95-year-old residents of the municipalities of Umeå, Dorotea, Malå, Sorsele, Storuman, and Vilhelmina in Sweden and Vaasa/Vasa, Korsholm/Mustasaari, Korsnäs/Ristitaipale, and Malax/Maalahti in Finland were invited to participate in the study. Umeå and Vaasa/Vasa are urban municipalities, and the other municipalities are rural. Swedish is the predominant language in all of the Swedish municipalities. In 2019 in Vaasa/Vasa, 70% of residents were Finnish speakers and 23% of residents were Swedish speakers; in the other participating municipalities in Finland, people spoke predominantly Swedish (Statistics Finland, 2020).

### Procedure

Potential participants were mailed letters of invitation and were contacted by telephone about one week later to obtain informed consent and schedule home visits. In cases of cognitive impairment, individuals’ next of kin were contacted and informed consent and participation were discussed. Educated and trained assessors (physicians, nurses, physiotherapists, and medical students) collected data using a guided questionnaire and assessment scales in participants’ homes.

### Participants

**Figure 1.**
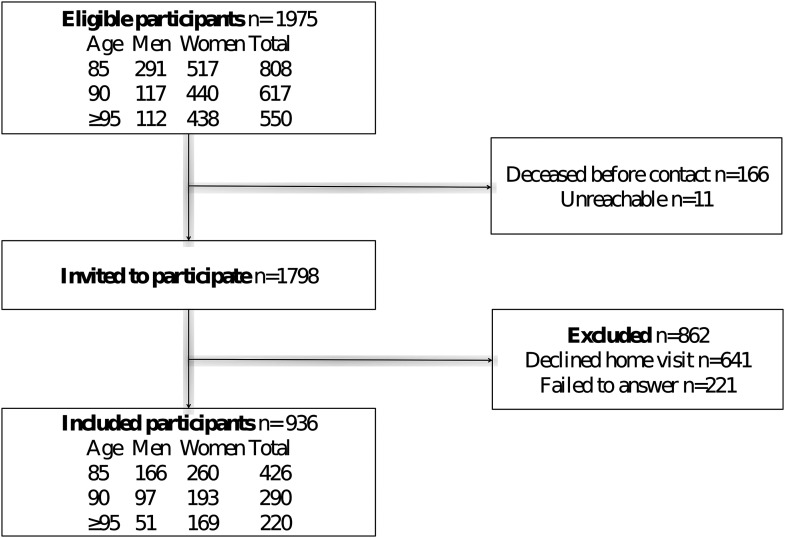
Flowchart of study participation.

A flowchart of study participation is shown in Figure 1. Participants in the present study met the inclusion criteria of the GERDA study and answered the question concerning plans for the future during home visits. Of 1,975 eligible participants, 166 died before being asked to participate and 11 were unreachable. Consequently, 1,798 people received invitations to participate in the GERDA. Eight hundred sixty-two people were excluded from the present study (641 declined home visits and 221 did not answer the question concerning plans for the future). Those who were excluded were older (mean age, 89.4 vs. 90.4 years; *p* < .001) and a larger proportion of them were women (66.5% vs. 75.6%; *p* < .001). Of those who participated in home visits but did not answer the question regarding plans for the future, 191 had dementia diagnoses and 30 were unable to answer the question for various other reasons, including other forms of cognitive impairment, inability to take a position, and interruption of the interview due to, for example, exhaustion. The remaining 936 individuals were included in the present study. For people who participated in the GERDA study more than once, first responses to the question about plans for the future were used. The follow-up period was 5 years, starting on the inclusion date and continuing until death, the end of the 5 years, or May 20, 2015 for those who had been included less than 5 years earlier.

### Measures

The existence of plans for the future was assessed using the question: “Do you have plans for the future?,” with the response options of “yes” and “no.” Information concerning diagnoses, medical conditions, and drug prescriptions was retrieved from the participants and their medical records from hospitals, general practitioners, and care institutions. In cases of cognitive impairment, participants’ next of kin and caregivers were asked to provide information when necessary. Data on death were retrieved from the Swedish Tax Agency, Finnish Population Register Center, medical records, and death certificates.

Participants’ height and weight were measured and their body mass indices (BMIs; kilograms/meters squared) were calculated. The 15-item Geriatric Depression Scale (GDS-15) was used to assess depressive symptoms (Sheikh & Yesavage, 1986). Scores range from 0 to 15 and higher scores indicate more severe depressive symptoms. The GDS-15 has been reported to detect clinical depression with high sensitivity and specificity in very old people (de Craen et al., 2003), very old people with cognitive impairment (Conradsson et al., 2013), and people living in care institutions (Smalbrugge et al., 2008). The Mini-Mental State Examination (MMSE) was used to assess cognitive function (Folstein et al., 1975; Tombaugh & McIntyre, 1992). Scores range from 0 to 30 and higher scores indicate better cognitive functioning. The 10-item version of the Barthel Activities of Daily Living (ADL) Index was used to measure dependence in personal ADL. Scores on this well-established, validated scale range from 0 to 20, with higher scores indicating greater independence (Collin et al., 1988; Sainsbury et al., 2005). Morale was assessed using the 17-item Philadelphia Geriatric Center Morale Scale (PGCMS), for which scores range from 0 to 17, and higher scores indicate higher morale (Lawton, 1975). The Swedish version of the PGCMS has been shown to be feasible for the assessment of morale in very old people and to have satisfactory psychometric properties (Niklasson et al., 2015). Nutritional status was assessed using the Mini Nutritional Assessment (MNA), which has been validated for the identification of malnutrition in older patients (Vellas et al., 1999). MNA scores range from 0 to 30 and higher scores indicate better nutritional status. Edentulous patients were identified using data on dental prostheses.

### Statistical Analyses

Baseline variables thought to be associated with having plans for the future were identified based on previous research, clinical experience, and scientific interest. The *χ*^2^ test and Student's *t* test were used to examine group differences. *p* values < .05 were considered to be significant. Total GDS-15 scores were imputed for incomplete responses with ≥10 of 15 items answered by dividing each incomplete score by the number of questions answered and then multiplying by 15 (Shrive et al., 2006). GDS-15 responses with <10 items answered were treated as missing data. In accordance with previous research, PGCMS scores were imputed for incomplete responses with ≥12 of 17 items answered by dividing each incomplete score by the number of questions answered and then multiplying by 17 (Näsman et al., 2019; Niklasson et al., 2017). PGCMS responses with <12 items answered were treated as missing data.

Variables associated significantly with having plans for the future in univariate analyses were entered into a multivariate logistic regression model to examine the independence of these associations. PGCMS scores were excluded from the models due to multicollinearity with GDS-15 scores, and country of residence was excluded due to multicollinearity with language. Language and country of residence were associated independently with having plans for the future when included separately. Multivariate logistic regression was performed for the total sample and for subcohorts of women and men. Variables associated significantly with having plans for the future in the subcohorts were included together with age, which was associated significantly with having plans for the future in women, but not in men. Due to a lack of power in the Finnish-speaking subcohort, no multivariate logistic regression was performed for language subcohorts.

Cox proportional-hazard regression models were used to analyze survival in relation to having plans for the future. The log-rank test was used to compare survival distributions between those who did and did not have plans for the future. Multivariate Cox regression was performed to analyze the association between having plans for the future and 5-year survival, adjusted for potentially confounding factors (*p* < .15 in multivariate logistic regression), age, and gender. The final model included having plans for the future, age, gender, language, community-dwelling status, stroke history, edentulism, and GDS-15 score. The assumption of hazard proportionality was tested using a Schoenfeld residual-based test (Kleinbaum & Klein, 2012), which indicated that no potential confounder was time dependent. The SPSS Statistics software (Version 24.0 for Macintosh; IBM Corporation, Armonk, NY, USA) was used for the statistical analyses.

## Results

### Characteristics of the Study Population

The participants were aged 84–104 years (mean, 89.4 years). Women comprised 66.5% of the study population; 65.1% of participants lived in Sweden and 84.9% of participants spoke Swedish. In the total sample, 18.6% of participants had plans for the future; 22.3% of 85-year-olds, 18.3% of 90-year-olds, and 11.8% of ≥95-year-olds had such plans. The difference between 90- and 95-year-olds was significant (*p* = .046).

### Factors Associated With Having Plans for the Future

In the total sample, having plans for the future was associated univariately with being male, speaking Swedish, living in the community, being dentate, having a higher MMSE score, having a higher PGCMS score, having a lower GDS-15 score, having a higher MNA score, having a higher Barthel ADL Index score, living with a partner, being younger, living in Sweden, having living children, having a higher level of education, and not having had a stroke. It was not associated significantly with being satisfied with one's personal finances or being able to make ends meet (Table 1). In the multivariate logistic regression analysis, speaking Swedish, being dentate, and living in the community were associated independently with having plans for the future (Table 2).

**Table 1.** Characteristics of the Study Population by Having and Not Having Plans for the Future.

Variable	Total	Plans (n = 174)	No Plans (n = 762)	p-value
Age	936	88.4 ± 4.1	89.6 ± 4.6	*.*002
Gender, woman	622 (66.5)	96 (55.2)	526 (69.0)	<.001
Education, more than 7 years (n = 896)	286 *(*31.9)	66 *(*39.3)	220 *(*30.2)	*.*023
Country of residence, Sweden	609 *(*65.1)	128 *(*73.6)	481 *(*63.1)	*.*009
Language, Swedish	795 (84.9)	163 (93.7)	632 (82.9)	<.001
Living children (n = 905)	795 *(*87.8)	159 *(*93.0)	636 *(*86.6)	*.*022
Living in a rural community	332 *(*35.5)	54 *(*31.0)	278 *(*36.5)	*.*175
Living in the community (n = 935)	686 (73.4)	148 (85.5)	538 (70.6)	<.001
Living with a partner (n = 929)	208 *(*22.4)	56 *(*32.2)	152 *(*20.1)	*.*001
Satisfied with personal finances (n = 860)	822 *(*95.6)	159 *(*96.4)	663 *(*95.4)	*.*587
Making ends meet (n = 842)	726 *(*86.2)	138 *(*84.7)	588 *(*86.6)	*.*520
Having had a stroke	179 *(*19.1)	23 *(*13.2)	156 *(*20.5)	*.*028
Chronic pulmonary disease	152 *(*16.2)	27 *(*15.5)	125 *(*16.4)	*.*775
Heart failure	282 *(*30.1)	47 *(*27.0)	235 *(*30.8)	*.*321
Myocardial infarction in the last 5 years	82 *(*8.8)	20 *(*11.5)	62 *(*8.1)	*.*157
Diabetes mellitus	182 *(*19.4)	38 *(*21.8)	144 *(*18.9)	*.*376
Sleeping disorder	363 *(*38.8)	75 *(*43.1)	288 *(*37.8)	*.*195
Pain in the last week (n = 928)	515 *(*55.5)	98 *(*56.6)	417 *(*55.2)	*.*735
Edentulous (n = 845)	447 (52.9)	62 (39.5)	385 (56.0)	<.001
Barthel ADL Index	933	18.6 ± 3.0	17.6 ± 4.0	*.*001
Body Mass Index	918	25.6 ± 3.7	26.0 ± 4.3	*.*319
Geriatric Depression Scale-15	905	2.7 ± 2.4	3.7 ± 2.5	<.001
Mini-Mental State Examination	928	24.2 ± 4.9	22.5 ± 5.4	<.001
Philadelphia Geriatric Centre Morale Scale	726	13.0 ± 3.0	12.1 ± 3.0	.002
Mini Nutritional Assessment	888	25.5 ± 3.3	24.2 ± 3.2	<.001

*Note.* Data are presented as *n* (%) or mean ± standard deviation. Differences between groups were examined using the *χ*^2^ test and Student's *t* test. ADL = activities of daily living.

**Table 2.** Multivariate Associations With Having Plans for the Future.

Variable	Wald.	OR	95% CI	*p*-value
Age	0.897	0.975	0.926–1.027	.344
Gender	3.210	1.459	0.965–2.206	.073
Education	1.139	0.796	0.523–1.211	.286
Language, Swedish	13.369	0.239	0.111–0.515	<.001
Living with a partner	1.146	1.290	0.809–2.054	.284
Living in the community	3.938	0.500	0.252–0.991	.047
Living children	1.447	0.662	0.339–1.296	.229
Having had a stroke	2.705	0.620	0.351–1.096	.100
Edentulous	5.430	0.619	0.413–0.927	.020
Barthel ADL Index	1.611	0.946	0.868–1.031	.204
Mini-Mental State Examination	1.272	1.031	0.978–1.087	.259
Geriatric Depression Scale-15	3.215	0.917	0.833–1.008	.073
Mini Nutritional Assessment	1.607	1.055	0.971–1.145	.205

*Note:* The multivariate logistic regression model included variables associated significantly with having plans for the future in univariate analyses (*p* < .05). OR = odds ratio; CI = confidence interval; ADL = activities of daily living.

### Variance Related to Gender, Country, and Language

Several significant differences between women and men were observed; 15.4% of women and 24.8% of men had plans for the future. The women were older than the men and constituted larger proportions of Finnish-speaking than of Swedish-speaking people and of people living in Finland than of those living in Sweden. Fewer women than men lived in rural communities, communities (rather than residential care facilities), and with partners, and felt that they were able to make ends meet. More women than men had sleeping disorders, heart failure, and recent pain and were edentulous; women had a lower prevalence of chronic pulmonary disease and lower mean Barthel ADL Index, PGCMS, MNA, and MMSE scores (Table 3).

**Table 3.** Baseline Characteristics of Study Participants by Gender.

Variable	Total	Women (*n* = 622)	Men (*n* = 314)	*p*-value
Plans for the future	174	96 (15.4)	78 (24.8)	<.001
Age		89.8 ± 4.7	88.5 ± 4.0	<.001
Education, more than 7 years (*n* = 896)	286	184 (30.8)	102 (34.1)	.319
Language, Swedish	795	512 (82.3)	283 (90.1)	.002
Country of residence, Sweden	609	390 (62.7)	219 (69.7)	.033
Living children (*n* = 905)	795	527 (87.5)	268 (88.4)	.693
Living in a rural community	332	195 (31.4)	137 (43.6)	<.001
Living in the community	686	431 (69.3)	255 (81.5)	<.001
Living with a partner (*n* = 929)	208	65 (10.5)	143 (45.8)	<.001
Satisfied with personal finances (*n* = 860)	822	538 (95.9)	284 (95.0)	.533
Making ends meet (*n* = 842)	726	460 (84.4)	266 (89.6)	.038
Having had a stroke	179	120 (19.3)	59 (18.8)	.853
Chronic pulmonary disease	152	85 (13.7)	67 (21.3)	.003
Heart failure	282	204 (32.8)	78 (24.8)	.012
Myocardial infarction in the last 5 years	82	51 (8.2)	31 (9.9)	.393
Diabetes mellitus	182	123 (19.8)	59 (18.8)	.719
Sleeping disorder	363	272 (43.7)	91 (29.0)	<.001
Pain in the last week (*n* = 928)	515	367 (59.5)	148 (47.6)	.001
Edentulous	447	318 (56.7)	129 (45.4)	.002
Barthel ADL Index	933	17.4 ± 4.0 (*n* = 619)	18.4 ± 3.3 (*n* = 314)	<.001
Body Mass Index	918	26.0 ± 4.4 (*n* = 607)	25.7 ± 3.7 (*n* = 311)	.266
Geriatric Depression Scale-15	905	3.6 ± 2.5 (*n* = 599)	3.3 ± 2.6 (*n* = 306)	.121
Mini-Mental State Examination	928	22.5 ± 5.4 (*n* = 617)	23.3 ± 5.1 (*n* = 311)	.036
Mini Nutritional Assessment	888	24.2 ± 3.3 (*n* = 592)	24.9 ± 3.2 (*n* = 296)	.004
Philadelphia Geriatric Center Morale Scale	726	12.0 ± 3.1 (*n* = 475)	12.7 ± 2.9 (*n* = 251)	.002

*Note.* Data are presented as *n* (%) or mean *± *standard deviation. Total numbers are presented in parentheses for variables with missing data. Differences between groups were examined using the *χ*^2^ test and Student's *t* test. ADL = activities of daily living.

Among women, factors associated univariately with having plans for the future were younger age (*p* = .031), speaking Swedish (*p* = .020), lower GDS-15 score (*p* = .008), higher MMSE score (*p* = .008), higher Barthel ADL Index score (*p* = .001), being dentate (*p* = .001), higher MNA score (*p* < .001), living in the community (*p* = .012), having living children (*p* = .048), and PGCMS score (*p* = .034, excluded from the model due to multicollinearity with GDS-15 score). The multivariate logistic regression model revealed independent associations of speaking Swedish and being dentate with having plans for the future (Table 4).

**Table 4.** Multivariate Associations with Having Plans for the Future in Women.

Variable	Wald.	OR	95% CI	*p*-value
Age	0.068	0.992	0.933–1.054	.794
Language, Swedish	6.053	0.329	0.136–0.798	.014
Living in the community	0.038	0.927	0.428–2.006	.846
Geriatric Depression Scale-15	0.772	0.948	0.840–1.069	.380
Mini-Mental State Examination	0.000	1.000	0.939–1.066	.998
Barthel ADL Index	1.528	1.082	0.955–1.226	.216
Edentulous	6.297	0.521	0.313–0.867	.012
Living children	1.618	0.555	0.224–1.375	.203
Mini Nutritional Assessment	1.496	1.067	0.961–1.185	.221

*Note.* The multivariate logistic regression model included variables associated significantly with having plans for the future among women in univariate analyses (*p* < .05). OR = odds ratio; CI = confidence interval; ADL = activities of daily living.

Among men, speaking Swedish (*p* = .013), lower GDS-15 score (*p* < .001), higher MMSE score (*p* = .015), higher MNA score (*p* = .024), living in the community (*p* = .005), and living in an urban community (*p* = .004) were associated univariately with having plans for the future and entered into the multivariate logistic regression model. Age (*p* = .129) was also included in the analysis. The multivariate logistic regression model revealed independent associations of speaking Swedish, living in an urban community, and lower GDS-15 scores with having plans for the future (Table 5).

**Table 5.** Multivariate Associations With Having Plans for the Future in Men.

Variable	Wald.	OR	95% CI	*p*-value
Age	0.586	0.968	0.890–1.053	.444
Language, Swedish	7.571	0.121	0.027–0.545	.006
Living in the community	2.305	0.443	0.155–1.267	.129
Geriatric Depression Scale-15	4.923	0.850	0.737–0.981	.027
Mini-Mental State Examination	1.338	1.046	0.969–1.128	.247
Mini Nutritional Assessment	0.007	1.005	0.899–1.123	.934
Living in a rural community	5.670	0.481	0.263–0.878	.017

*Note.* The multivariate logistic regression model included variables associated significantly with having plans for the future among men in univariate analyses (*p* < .05). OR = odds ratio; CI = confidence interval; ADL = activities of daily living.

More participants living in Sweden than in Finland (21% vs. 14%; *p* = .009) and more Swedish-speaking than Finnish-speaking Finns (19% vs. 8%; *p* = .005) had plans for the future. Compared with Swedish participants, Finnish participants were more often edentulous, more often lived in the community, less often satisfied with their personal finances, and more often had difficulty making ends meet; they had higher levels of education, higher BMIs, higher MMSE scores, a lower prevalence of stroke, lower GDS-15 scores, a lower prevalence of sleeping disorders, higher Barthel ADL Index scores, a greater proportion of women, and a higher prevalence of diabetes mellitus. Several differences were observed between the Swedish- and Finnish-speaking subcohorts of Finnish residents. Fewer Finnish- than Swedish-speaking Finns and Swedes had plans for the future (*p* = .005). A large proportion of the Finnish-speaking cohort lived in urban communities, and women comprised a greater proportion of Finnish-speaking than of Swedish-speaking Finns. Finnish-speaking Finns had higher GDS-15 scores, lower MNA scores, lower prevalence of difficulty making ends meet, lower Barthel ADL Index scores, higher prevalence of heart failure, higher prevalence of myocardial infarction, and lower prevalence of having living children than did Swedish-speaking Finns (Table 6).

**Table 6.** Baseline Characteristics of Study Participants by Country of Residence and, in Finland, by Language.

Variable	Swedes (*n* = 609)	Finns (*n* = 327)	*p*-value^a^	Swedish-speaking Finns (*n* = 186)	Finnish-speaking Finns (*n* = 141)	*p*-value^b^
Plans for the future	128 (21.0)	46 (14.1)	.009	35 (18.8)	11 (7.8)	.005
Age	89.3 ± 4.4	89.5 ± 4.7	.638	89.4 ± 4.6	89.6 ± 4.8	.739
Gender, woman	390 (64.0)	232 (70.9)	.033	122 (65.6)	110 (78.0)	.014
Education, more than 7 years	146 (25.6)	140 (43.1)	<.001	87 (47.0)	53 (37.9)	.098
Living children	515 (87.9)	280 (87.8)	.962	163 (91.1)	117 (83.6)	.043
Living in a rural community	220 (36.1)	112 (34.3)	.568	103 (55.4)	9 (6.4)	<.001
Living in the community	430 (70.7)	256 (78.3)	.013	151 (81.2)	105 (74.5)	.145
Living with a partner	468 (77.6)	253 (77.6)	.999	141 (76.2)	112 (79.4)	.490
Satisfied with personal finances	554 (96.9)	268 (93.1)	.011	150 (90.9)	118 (95.9)	.097
Making ends meet	493 (88.4)	233 (82.0)	.012	117 (73.1)	116 (93.5)	<.001
Having had a stroke	140 (23.0)	39 (11.9)	<.001	20 (10.8)	19 (13.5)	.452
Chronic pulmonary disease	99 (16.3)	53 (16.2)	.985	28 (15.1)	25 (17.7)	.515
Heart failure	186 (30.5)	96 (29.4)	.707	43 (23.1)	53 (37.6)	.004
Myocardial infarction in the last 5 years	58 (9.5)	24 (7.3)	.260	7 (3.8)	17 (12.1)	.004
Diabetes mellitus	107 (17.6)	75 (22.9)	.048	41 (22.0)	34 (24.1)	.659
Sleeping disorder	255 (41.9)	108 (33.0)	.008	62 (33.3)	46 (32.6)	.893
Pain in the last week	337 (55.7)	178 (55.1)	.862	98 (53.0)	80 (58.0)	.372
Edentulous	274 (48.5)	173 (61.8)	<.001	81 (58.3)	92 (65.2)	.230
Barthel ADL Index	17.5 ± 4.1	18.1 ± 3.3	.023	18.6 ± 2.5	17.5 ± 4.0	.002
Body Mass Index	25.5 ± 4.1	26.6 ± 4.1	<.001	26.6 ± 4.1	26.8 ± 4.2	.671
Geriatric Depression Scale-15	3.6 ± 2.6	3.2 ± 2.4	.022	2.8 ± 2.3	3.8 ± 2.5	<.001
Mini-Mental State Examination	22.2 ± 5.5	23.9 ± 4.8	<.001	23.8 ± 4.6	24.0 ± 5.0	.835
Mini Nutritional Assessment	24.4 ± 3.4	24.5 ± 3.0	.752	25.2 ± 2.7	23.8 ± 3.0	<.001
Philadelphia Geriatric Center Morale Scale	12.2 ± 3.0	12.4 ± 3.4	.499	13.0 ± 3.4	11.9 ± 3.3	.059

*Note.* Data are presented as *n* (%) or mean ± standard deviation. Differences between groups were examined using the *χ*^2^ test and Student's *t* test. ADL = activities of daily living.

a Swedes versus Finns.

b Swedish-speaking versus Finnish-speaking Finns.

### Survival

**Figure 2.**
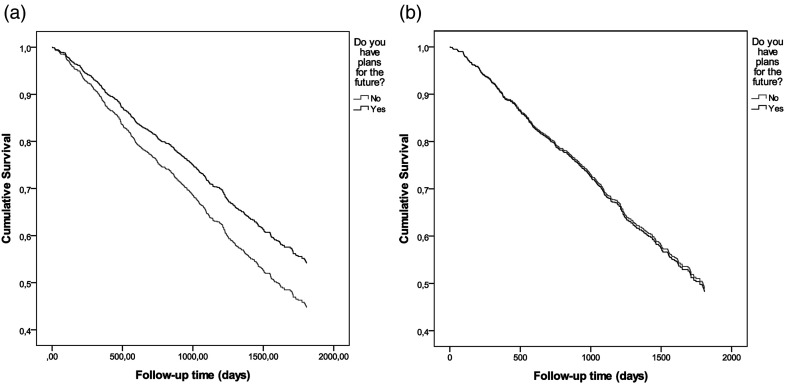
Survival curves according to the possession of plans for the future based on an unadjusted Cox proportional-hazard regression model (a) and on a Cox proportional-hazard regression model adjusted for age, gender, language, living in the community, stroke history, edentulism, and geriatric depression scale score (b).

The 5-year mortality rate was significantly lower among participants with than among those without plans for the future, according to a log-rank test (*p* = .036) and univariate Cox proportional-hazard regression model (*p* = .037; hazard ratio [HR] 0.763, 95% confidence interval [CI] 0.592–0.983; Figure 2a). In the multivariate model, having plans for the future was not associated significantly with survival (Figure 2b; *p* = .893, HR 0.981, 95% CI 0.738–1.303). In the final model, survival was associated significantly with younger age (*p* < .001; HR 1.107, 95% CI 1.082–1.132), living in the community (*p* < .001; HR 1.700, 95% CI 1.350–2.140), female gender (*p* < .001; HR 1.696, 95% CI 1.363–2.110), and lower GDS-15 score (*p* < .001; HR 1.072, 95% CI 1.031–1.114).

## Discussion

The majority (81%) of very old people did not have plans for the future. Speaking Swedish, being dentate, and living in the community were the only factors associated independently with having such plans. Although gender was not associated independently with having plans for the future, some factors differed between genders. An independent association with being dentate was observed among women, but not men; independent associations with having fewer depressive symptoms and living in an urban community were observed among men, but not women. Larger proportions of people in Sweden than in Finland, and of Swedish-speaking than of Finnish-speaking Finns, had plans for the future. Having plans for the future was associated with increased survival in a univariate analysis, but not in an analysis adjusted for potential confounders.

### Prevalence

In the present study, 81% of very old people did not have plans for the future. Among 85-year-olds, the prevalence of having plans for the future was 22.3%, in agreement with the previously reported prevalence of 21% for the same age group (Pitkälä et al., 2004). Such findings have been suggested to reflect a realistic outlook regarding the future, with no making of plans in the face of deteriorating physical and psychological health, as well as the inherent unpredictability of life in very old age (Baltes & Smith, 2003; Kotter-Grühn & Smith, 2011).

### Factors Associated With Having Plans for the Future

In the full study population, speaking Swedish, being dentate, and living in the community were associated independently with having plans for the future. The possession of plans for the future differed significantly between participants in Sweden and Finland, but the difference between Swedish-speaking and Finnish-speaking Finns was greater, suggesting that language and/or associated cultural differences are more important than country of residence. These findings contradict those of a survey conducted with a younger population of 65- and 75-year-olds in the same regions of Sweden and Finland (Fagerström, 2010). Several studies have demonstrated that Swedish-speaking Finns have more social capital than do Finnish-speaking Finns, regardless of socioeconomic factors and level of education, and that this greater social capital is associated with health benefits (Hyyppä & Mäki, 2001; Nyqvist et al., 2008). This factor may explain the language-based differences in having plans for the future observed in the present study. Participants in the present study were born between 1902 and 1925, meaning that the Finnish study population grew up during the Finnish civil war in 1918, the Winter War, and the Continuation War during World War II. Trauma related to growing up in wartime could influence individuals’ outlooks on the future. War-related traumatic experiences in childhood and adolescence have been associated with adverse outcomes in old age, including traumatic symptoms, post-traumatic stress disorder (Glaesmer et al., 2010), and medical conditions (Glaesmer et al., 2011), which may explain the difference between people living in Sweden and Finland observed in this study. To better understand these differences, further research with the Finnish-speaking cohort is needed.

Edentulism may be an expression of poor oral health and is often a consequence of periodontitis (Petersen & Ogawa, 2012). The association between edentulism and having no plans for the future observed in this study could be explained by several factors. For example, poor oral health is associated with lower socioeconomic status (Olofsson et al., 2018; Petersen & Yamamoto, 2005; Starr et al., 2008; Wamala et al., 2006). However, in the very old cohort of the present study, socioeconomic factors were not associated independently with having plans for the future. Poor oral health has also been associated with poor general health (Petersen & Yamamoto, 2005), less social participation (Rodrigues et al., 2012), and lower self-esteem (Starr et al., 2008), and may lead to inadequate nutritional status among old people (Ervin & Dye, 2009; Walls et al., 2000). All of these aspects may have contributed to our finding; further investigation is needed.

Factors predicting old people's admission to nursing homes include older age (Gaugler et al., 2007; Luppa et al., 2010), low self-rated health status (Luppa et al., 2010), functional impairment (Gaugler et al., 2007; Hajek et al., 2015; Luppa et al., 2010), cognitive impairment (Gaugler et al., 2007; Hajek et al., 2015; Luppa et al., 2010), dementia diagnosis (Luppa et al., 2010), larger numbers of prescriptions (Luppa et al., 2010), prior nursing home residence (Gaugler et al., 2007; Luppa et al., 2010), poor informal support (Gaugler et al., 2007; Luppa et al., 2010), lack of socioeconomic resources (e.g., home ownership, single marital status), not having a spouse or being widowed (Gaugler et al., 2007; Hajek et al., 2015; Luppa et al., 2010), and severe depression (Hajek et al., 2015). Although these factors were not associated independently with having plans for the future in the present study, apart from higher GDS-15 scores among men, their presence may reflect substantially deteriorating health and function resulting in a lack of ability to care for oneself, which indirectly may explain the observed association between living in the community and having plans for the future.

Our univariate analysis revealed that more men than women had plans for the future, in agreement with previous findings (Fagerström, 2010; Tilvis et al., 2012). However, male gender was not associated independently with having plans for the future in the multivariate logistic regression analysis, which also is in line with previous findings (Eloranta et al., 2012). Factors other than gender may better explain the observed association.

In our multivariate logistic regression analyses, different factors were associated with having plans for the future among women and men, apart from speaking Swedish, which showed independent associations in both cohorts. Among women, being dentate was the only other independently associated variable; among men, having fewer depressive symptoms and living in an urban community were associated independently with having plans for the future. A meta-analysis showed that depressed people have difficulty imagining specific positive futures as a result of overgenerality, and that this effect is stronger among men than women (Gamble et al., 2019), which may explain the association of having plans for the future with fewer depressive symptoms in men in the present study. The effect of urban living among men may be related to various factors. Loneliness and social isolation could be more prevalent in rural than in urban communities due to factors such as lower population densities, longer traveling distances, and less access to local services, which may affect people's ability to participate in social activities, in turn affecting very old people's planning for the future. However, reports regarding such associations have been inconsistent. Studies conducted in Finland have shown that loneliness is more common in rural than in urban areas (Savikko et al., 2005) and that rural residence is associated univariately, but not multivariately, with loneliness and social isolation (Routasalo et al., 2006). In contrast, a study conducted in Canada showed that social isolation, but not loneliness, is more prevalent in urban than in rural areas; this association, however, was significant in a univariate, but not multilevel logistic regression, analysis (Menec et al., 2019). These findings suggest that loneliness and social isolation in rural areas cannot explain the association with having plans for the future observed in this study. A study conducted in Sweden revealed that more old people in urban areas than those in rural areas live close to their children, which may be a result of the weakening of family networks in rural areas due to migration patterns (Lundholm, 2015), which in turn may affect planning for the future among very old people who are dependent on such family networks. However, the factors underlying the observation of these associations in men, but not in women, remain unclear; further investigation is needed.

In univariate analysis, the proportion of people with plans for the future was lower among older participants, in agreement with previous findings (Eloranta et al., 2012; Fagerström, 2010; Kotter-Grühn & Smith, 2011; Pitkälä et al., 2004). In one study, age was associated significantly with having plans for the future during a 15-year follow-up period in an analysis adjusted for gender and living arrangement (Eloranta et al., 2012). The findings of the present study suggest that not chronological age, but potentially age-related factors included in the multivariate model (e.g., edentulism) are associated with having no plan for the future. A similar finding was reported in a previous study (Hoppmann et al., 2017).

### Association Between Having Plans for the Future and Survival

Although having plans for the future was not associated independently with increased survival in the multivariate Cox proportional-hazard regression analysis, this association was significant in univariate analysis, suggesting that such planning is a marker of increased survival. In our Cox proportional-hazard regression model, younger age, female gender, living in the community, and lower GDS-15 scores best explained the impact of having plans for the future on survival. In a previous study, adjusted analysis revealed a significant association between having plans for the future and survival (Tilvis et al., 2012). However, participants in that study were younger than those participating in the present study sample (mean age, 81 vs. 89.4 years). Any independent association of having plans for the future with a survival benefit may weaken with advancing age.

### Study Strengths and Limitations

Data for this study were collected during home visits, and the only inclusion criteria were based on age and place of residence. Thus, people diagnosed with dementia, those with multimorbidity, and those living in nursing homes were included, which is the study's strength. Despite the broad inclusion of participants, the sample was not representative of 85-, 90-, and ≥95-year-olds in the studied geographical area, as the excluded group differed from the study participants in terms of age and gender. However, as the difference in age between included and excluded people was relatively small, the results may still be valid for very old people, and as analyses have been performed in the gender subcohorts separately, the results may also be valid for these subcohorts. As a large proportion of those who participated in home visits but did not answer the question about having plans for the future were diagnosed with dementia, the study results may be less representative of this group. A limitation of this study is the use of a single dichotomous question to assess plans for the future; this concept is complex, and the assessment could have been more nuanced. Furthermore, the concept of having plans for the future was not defined; participants responded based on their interpretations of the question, which may have differed among individuals. The limited size of the Finnish-speaking subcohort is another limitation, as some subgroup analyses could not be performed. As the findings suggest the presence of differences related to cultural and linguistic factors, further research is needed to better understand such differences.

## Conclusion

The majority of very old people did not have plans for the future. Speaking Swedish, being dentate, and living in the community were associated with having such plans. Previously reported gender- and age-related differences were not confirmed in the present study but rather explained by health and social factors. The study revealed differences in factors associated with having plans for the future between women and men and in relation to cultural and linguistic differences between Swedish and Finnish people, Swedish-speaking Finns, and Finnish-speaking Finns in Finland, which should be examined more closely. Having plans for the future may also be a marker of better survival in very old people.
